# General Model for COVID-19 Spreading With Consideration of Intercity Migration, Insufficient Testing, and Active Intervention: Modeling Study of Pandemic Progression in Japan and the United States

**DOI:** 10.2196/18880

**Published:** 2020-07-03

**Authors:** Choujun Zhan, Chi Kong Tse, Zhikang Lai, Xiaoyun Chen, Mingshen Mo

**Affiliations:** 1 South China Normal University Guangzhou China; 2 City University of Hong Kong Hong Kong Hong Kong; 3 Sun Yat-sen University Guangzhou China

**Keywords:** pandemic spreading, SEICR model, COVID-19, prediction, effect of intervention

## Abstract

**Background:**

The coronavirus disease (COVID-19) began to spread in mid-December 2019 from Wuhan, China, to most provinces in China and over 200 other countries through an active travel network. Limited by the ability of the country or city to perform tests, the officially reported number of confirmed cases is expected to be much smaller than the true number of infected cases.

**Objective:**

This study aims to develop a new susceptible-exposed-infected-confirmed-removed (SEICR) model for predicting the spreading progression of COVID-19 with consideration of intercity travel and the difference between the number of confirmed cases and actual infected cases, and to apply the model to provide a realistic prediction for the United States and Japan under different scenarios of active intervention.

**Methods:**

The model introduces a new state variable corresponding to the actual number of infected cases, integrates intercity travel data to track the movement of exposed and infected individuals among cities, and allows different levels of active intervention to be considered so that a realistic prediction of the number of infected individuals can be performed. Moreover, the model generates future progression profiles for different levels of intervention by setting the parameters relative to the values found from the data fitting.

**Results:**

By fitting the model with the data of the COVID-19 infection cases and the intercity travel data for Japan (January 15 to March 20, 2020) and the United States (February 20 to March 20, 2020), model parameters were found and then used to predict the pandemic progression in 47 regions of Japan and 50 states (plus a federal district) in the United States. The model revealed that, as of March 19, 2020, the number of infected individuals in Japan and the United States could be 20-fold and 5-fold as many as the number of confirmed cases, respectively. The results showed that, without tightening the implementation of active intervention, Japan and the United States will see about 6.55% and 18.2% of the population eventually infected, respectively, and with a drastic 10-fold elevated active intervention, the number of people eventually infected can be reduced by up to 95% in Japan and 70% in the United States.

**Conclusions:**

The new SEICR model has revealed the effectiveness of active intervention for controlling the spread of COVID-19. Stepping up active intervention would be more effective for Japan, and raising the level of public vigilance in maintaining personal hygiene and social distancing is comparatively more important for the United States.

## Introduction

### Background

The global spread of the coronavirus disease (COVID-19) has shown no sign of subsiding since its emergence in Wuhan, China, in December 2019 [1]. As of March 21, 2020, a total of 276,472 cases of COVID-19 infection have been confirmed in over 185 countries, with a death toll of 11,417 [2]. Different control strategies at different levels of stringency have been applied to slow the spread of the virus in different countries [3]. Although some countries have seen peaks of infected cases and have observed significant reductions in the number of new infections in the local communities [2,4], the spread has continued in many countries, and surges in infected cases have been observed in Europe, the United States, and Australia. Intercity travel has been found to be a contributing factor to the rapid spread of the virus [5,6]. Thus, effective models for describing the pandemic progression in different cities should take into consideration the volume of intercity travel [4,7]. Additionally, the virus spread from one country to another through the air transportation network [8-10]. Hence, population flow is expected to play an important role in the transmission of COVID-19, and travel restrictions would effectively slow the transmission of COVID-19 [11]. Furthermore, the rapid spread of the virus in a population has often been a result of delayed information or unawareness of the real situation in that population, despite the wide dissemination of information related to COVID-19 outbreaks in other parts of the world. The most notable information latency lies in the number of confirmed cases reported, which depends on the ability of the particular country or city to perform tests as well as the possible bureaucracy in the local system of reporting. Thus, the number of confirmed cases is almost certainly not the true number of infected individuals at any given time [12], and an improved model for predicting the spreading progression should incorporate the latency associated with the reporting system as well as the possible missing cases leading to delay and loss of information. The traditional susceptible-exposed-infectious-recovered (SEIR) model [13,14] thus has obvious shortfalls in describing the spreading dynamics of the COVID-19 pandemic. In this work, we attempt to fill the main gap between the number of confirmed cases and the actual number of infected cases. Specifically, in the proposed model, an infected individual may become a confirmed case and then recovered or removed. Moreover, an infected individual may also be recovered or removed without being confirmed as infected. In other words, the basic model proposed here is a susceptible exposed infected confirmed removed (SEICR) model, which has an additional state corresponding to an individual having been confirmed by the authority as being infected.

On the basis of an SEICR model, we developed a model incorporating intercity travel data that accounts for any increase or decrease in the number of exposed and infected individuals in a city due to intercity migration. Furthermore, the level of intervention in the form of travel restriction, regional lockdown, or other active control measures would profoundly influence the rapidity of the virus spread and the eventual number of infected cases. The model should, therefore, allow the level of active intervention to be included as a control parameter and produce the appropriate progression profile. A specific parameter was used to adjust the level of active intervention in the simulation of future progression profiles, which corresponds quantitatively to the increase in the number of individuals eventually infected due to an additional infected individual at any given time. In this work, we apply the model to study the COVID-19 spreading progression in Japan and the United States. Data of confirmed and recovered cases in 47 Japanese prefectures or regions (January 15 to March 20, 2020) and 50 US states plus Washington, DC (February 20 to March 20, 2020) were used for fitting with the model and retrieval of parameter values. The parameters found were then adjusted to produce future progression trajectories corresponding to the implementation of different levels of active intervention.

### Data

The World Health Organization has currently set the alert level of COVID-19 to the highest and has made data related to the pandemic available to the public in a series of situation reports as well as other formats [15]. Our data include the number of confirmed infected cases, the cumulative number of confirmed infected cases, the number of recovered cases, and death tolls for 47 individual prefectures and regions in Japan, from January 15 to March 20, 2020, and for 50 states and a federal district (Washington, DC) in the United States from February 20 to March 20, 2020. Data organized in convenient formats are also available elsewhere [12,16,17]. Moreover, the monthly intercity migration data for February 2020 are available from official statistics provided by the Japanese government [18] and are used as indicative migration strengths between prefectures or regions in Japan. For the United States, annual data for the volume of interstate travelers are available from the Census Bureau [19] and the Bureau of Transportation Statistics [20].

## Methods

### Migration-Data Augmented SEICR Model

**Figure 1:**
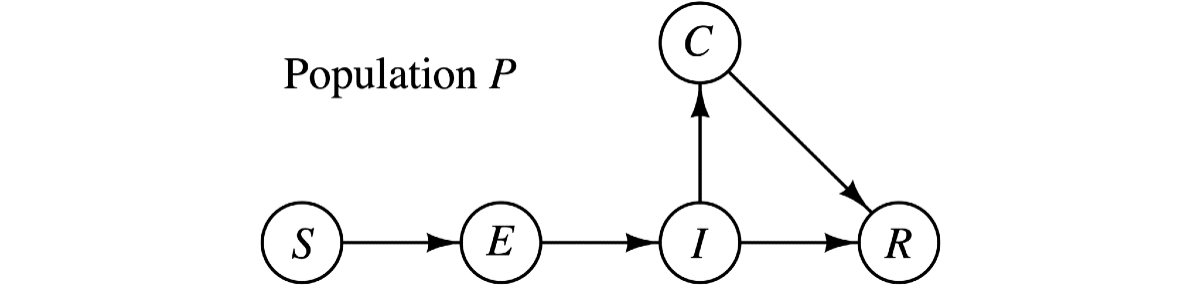
State transition flow chart. C: confirmed; E: exposed; I: infected; R: removed; S: susceptible.

In the proposed SEICR model, every individual would assume one of five possible states at any time, namely, susceptible (*S*), exposed (*E*), infected (*I*), confirmed (*C*), and recovered or removed (*R*). Compared to the traditional SEIR model [13,14], the new SEICR model has an additional *C* state, corresponding to an individual that has been confirmed by the authority as infected. Thus, not all infected individuals will become confirmed, and some infected individuals will transit to the recovered state without going through the confirmed state. For city or region *j*, the number of individuals in the five states are *S_j_*(*t*), *E_j_*(*t*), *I_j_*(*t*), *C_j_*(*t*), and *R_j_*(*t*) at time *t*. The transitions of the five states are illustrated in Figure 1.

In addition, *P_j_*(*t*) stands for the population of region *j*. Furthermore, to account for intercity movement, we introduce a migration strength, *m_ij_*(*t*), which represents an indicative volume of people moving from region *i* to region *j* at time *t* [4]. The augmented SEICR model is given as follows:


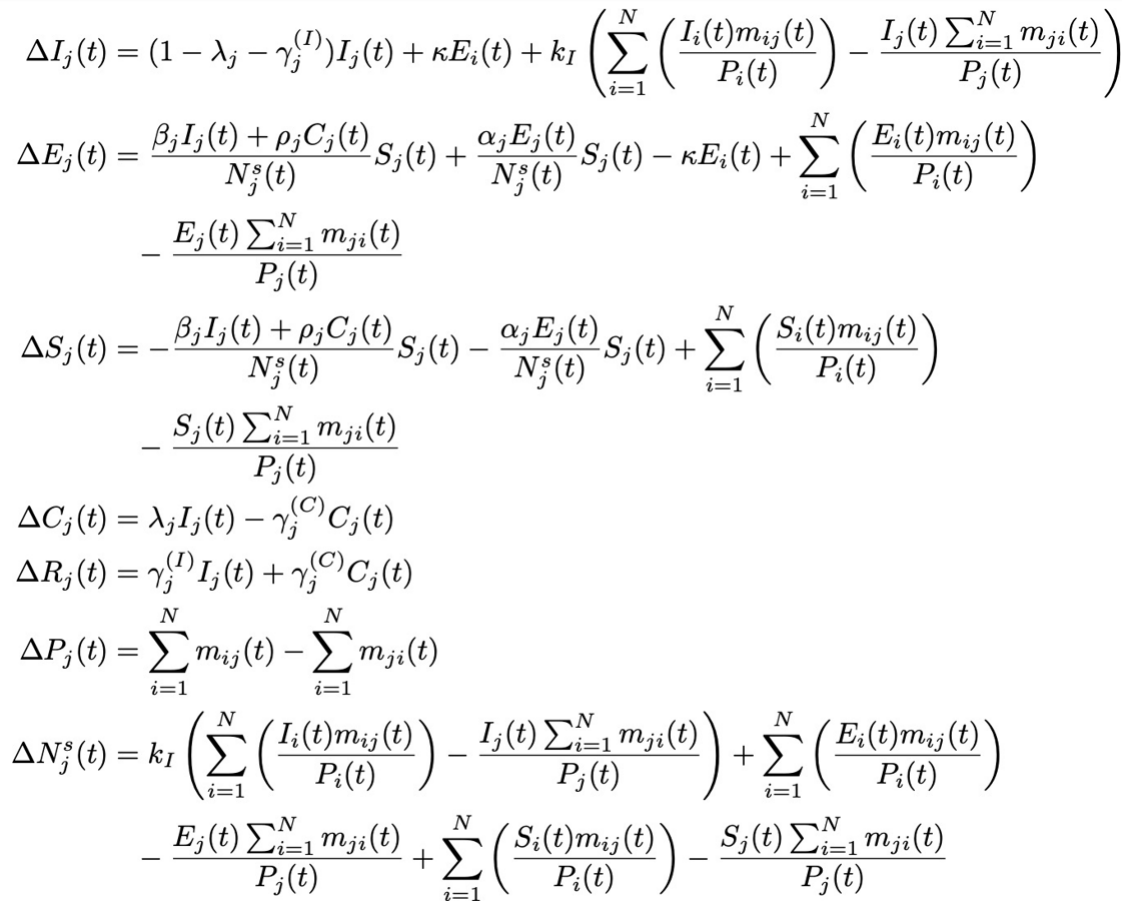
 **(1)**

Δ*I_j_*(*t*) = *I_j_*(*t* + 1) – *I_j_*(*t*), Δ*E_j_*(*t*) = *E_j_*(*t* + 1) – *E_j_*(*t*), Δ*S_j_*(*t*) = *S_j_*(*t* + 1) – *S_j_*(*t*), Δ*C_j_*(*t*) = *C_j_*(*t* + 1) – *C_j_*(*t*), Δ*R_j_*(*t*) = *R_j_*(*t* + 1) – *R_j_*(*t*), Δ*N_j_^s^*(*t*) = *N_j_^s^*(*t* + 1) – *N_j_^s^*(*t*), and Δ*P_j_*(*t*) = *P_j_*(*t* + 1) – *P_j_*(*t*).

The meaning of each parameter is given in Textbox 1. In addition, we assumed that the recovered and confirmed individuals would stay in region *j*.

In this SEICR model, the number of individuals eventually infected is set initially at *N_j_^s^*(*t*_0_) = *δ_j_P_j_* (*δ_j_* being constant), implying that some effective measures have been taken by the authorities to limit the upper bound of the susceptible population. Moreover, in the case of inactive or less effective intervention, or even unchecked spread, the growth in the number of infected cases will add to the eventual infected number. Hence, the number of eventually infected individuals should increase for each additional infected or exposed individual at time *t*. This is equivalent to adding an extra term (the boxed term below) to Δ*S_j_*(*t*) and Δ*N_j_^s^*(*t*). Furthermore, as the number of infected cases increases and approaches a saturating percentage *k_h_* (such as a herd-immunity condition), the spreading is expected to slow down significantly (ie, *α_j_* and *β_j_* will drop as *N_j_^s^* approaches *k_h_P_j_*, where 0<*k_h_*<1). Thus, we have:


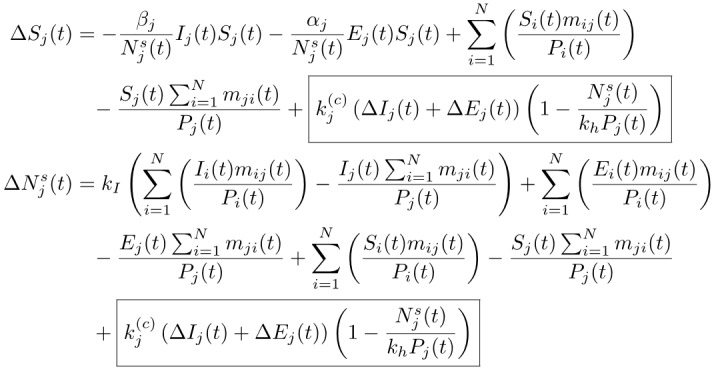
 **(2)**

*k_j_*^(c)^ is an inverse indicator of the level of active intervention implemented, and corresponds quantitatively to an increase in the number of eventual infected individuals for each additional infected or exposed individual in region *j*, and the added term in Δ*S_j_*(*t*) and Δ*N_j_^s^*(*t*) will approach zero as *N_j_^s^* → *k_h_P_j_*. The meanings of other parameters are given in Textbox 1. Again, the recovered and confirmed individuals are assumed to stay in region *j*.

The model given in (1) and (2) is general in the sense that it applies to populations with varied effectiveness levels of active intervention during the outbreak. To further facilitate the assessment of control measures implemented in region *j*, we defined the level of active intervention as:


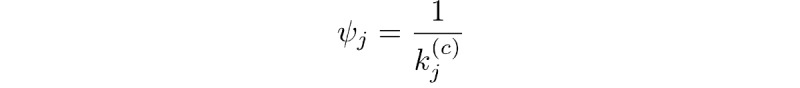
 **(3)**

Thus, if *ψ_j_*>1, the control measures are effective and the progression is limited such that *k_j_*^(c)^<1. The total number of eventually infected individuals is equal to 
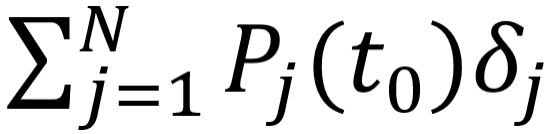
. However, in the case of less effective or ineffective control (ie, *ψ_j_*<1), infected and exposed individuals continue to spread the disease, and for each additional infected individual, there will be *k_j_*^(c)^ more eventual infected individuals, and the pandemic progresses until the number of infected cases reaches *k_h_P_j_*.

**Parameters of the migration-data augmented susceptible-exposed-infected-confirmed-removed model.**

***α_j_***

Rate of infecting a susceptible individual by an exposed individual in region *j*

***β_j_***

rate of infecting a susceptible individual by an infected individual in region *j*

***ρ_j_***

rate of infecting a susceptible individual by a confirmed individual in region *j*

***λ_j_***

confirmed rate of infected individuals in region *j*

***κ_j_***

rate of an exposed individual becoming infected

***γ_j_*^(*I*)^**

recovery rate of an infected but not confirmed individual in region *j*

***γ_j_*^(*C*)^**

recovery rate of a confirmed individual in region *j*

***k*_I_**

possibility of an infected individual moving from one region to another

***k_j_*^(c)^**

increase in number of individuals eventually infected for each additional infected or exposed individual in region *j*

***ψ_j_***

level of active intervention, *ψ_j_* = 1/*k_j_*^(c)^

***k_h_***

proportion of population infected achieving no further spreading (ie, absolute upper bound for *N_j_^s^* for all *j*)

***δ_j_***

initial percentage of eventual infected individuals in region *j*

***I_j_*_0_**

initial number of infected individuals in region *j*

***E_j_*_0_**

initial number of individuals in region *j*

***C_j_*_0_**

initial number of confirmed infected individuals in region *j*

### Parameter Identification

The model represented by (1) and (2) describes the dynamics of the pandemic propagation with consideration of human migration dynamics and the reality of insufficient testing that leads to confirmed infected cases being fewer than the actual infected cases. The parameters in (1) and (2) are unknown and to be estimated from historical data of *C* and *R*. We solve this parameter identification problem via constrained nonlinear programming with the objective of finding an estimated growth trajectory that fits the data. An estimated number of infected cases of each city can be generated from (1) and (2) with unknown set *θ_j_* given by:

*θ_j_* = {*α_j_*, *β_j_*, *γ_j_*, *δ_j_*, *λ_j_*, *γ_j_*^(^*^I^*^)^, *γ_j_*^(^*^C^*^)^, *k_j_*^(^*^c^*^)^, *I_j_*_,0_, *E_j_*_,0_}

**(4)**

*I_j_*_,0_(*t*) = *I_j_*(*t*_0_) and *E_j_*_,0_(*t*) = *E_j_*(*t*_0_) are the initial numbers of infected and exposed individuals in region *j*, and {*α_j_*, *β_j_*, *γ_j_*, *δ_j_*, *λ_j_*, *γ_j_*^(^*^I^*^)^, *γ_j_*^(^*^C^*^)^, *k_j_*^(^*^c^*^)^} are the model parameters of region *j*. Here, we assume that all confirmed cases are either quarantined or hospitalized and, hence, not infectious (ie, *ρ_j_*=0). The unknown set is then *Θ* = {*θ*_1_, *θ*_2_, …, *θ_K_*, *κ*, *k_I_*, *k_h_*}, which essentially has 8*K* + 3 unknowns, where *K* is the number of regions in the entire population under study. The identification of unknown parameters would require a considerable effort of computation.

Specifically, the parameter estimation problem can be formulated as the following constrained nonlinear optimization problem:


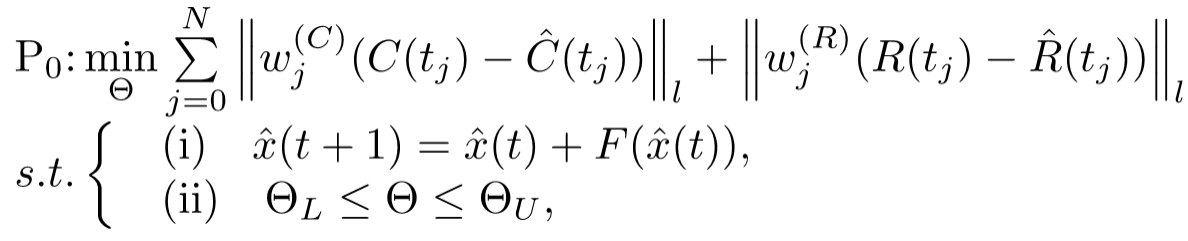
 **(5)**

*F*(.) represents the model given by (1) and (2), *ω_j_*^(^*^C^*^)^ and *ω_j_*^(^*^R^*^)^ are the weighting coefficients, and 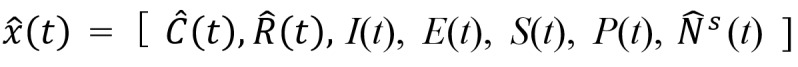 is the set of estimated variables, with unknown set Θ being bounded between Θ*_L_* and Θ*_U_*. In this work, an inverse approach is taken to find the unknown parameters and states by solving (5).

### Prediction

The model parameters characterize the spreading dynamics, and once the set of parameters has been identified using the previously mentioned optimization procedure, we may generate future progression profiles by using the same set of parameters. Moreover, we may also adjust some of the parameters to examine different possible scenarios, corresponding to varying levels of active intervention *ψ_j_* = 1/*k_j_*^(c)^, including travel restriction, mandatory quarantine, and other control measures. If the level of active intervention stays with the status quo, we will use the same value of *k_j_*^(c)^ for generating future progression profiles. Future paths under more active intervention can be predicted by reducing the value of *k_j_*^(c)^. In our study, by extending each simulation run to the forthcoming 200 days, we obtain a set of predicted progression profiles for each region in Japan and the United States. Moreover, different levels of active intervention can be assessed by adjusting parameter *k_j_*^(c)^ relative to the values found in each candidate set. For instance, by reducing *k_j_*^(c)^ and rerunning the simulation, we may assess the effect of tightening the control measures. In particular, we will consider three levels of active intervention: (1) staying with the status quo, corresponding to the same value of *ψ_j_* or *k_j_*^(c)^; (2) 2-fold step-up of active intervention, corresponding to 2*ψ_j_* or 0.5*k_j_*^(c)^; and (3) 4-fold step-up of active intervention, corresponding to 4*ψ_j_* or 0.25*k_j_*^(c)^

The pandemic progression profiles of 47 Japanese prefectures or regions were examined. We perform data fitting of the model, described by (1) and (2), using historical daily data of confirmed and recovered cases. For the United States, the pandemic progression profiles of 50 states and a federal district were examined. We again performed data fitting of the model using historical daily data of confirmed and recovered cases from February 20 to March 20, 2020, and obtained 100 candidate sets of parameters that satisfy the fitting criteria.

The level of public vigilance in exercising personal protective measures can also be incorporated in our model through adjusting infection rates *α_j_* and *β_j_*. We can, therefore, assess the combined effectiveness of active intervention and practicing protective measures in controlling the pandemic. Here, we varied *α_j_*, *β_j_*, and *k_j_*^(c)^ from 10% to 100% of the originally identified values in 10 intervals, corresponding to 10 different levels of public vigilance and active intervention by the authorities. In particular, we assess *α_j_* and *β_j_* as one property and *k_j_*^(c)^ as another (ie, varying *α_j_* and *β_j_* in synchrony). Specifically, for each candidate parameter set, we perform 100 simulation runs for each combination of *α_j_*, *β_j_*, and *k_j_*^(c)^, where *α_j_*, *β_j_*, and *k_j_*^(c)^ vary from 10% to 100% of the original values in 10 steps. We then investigate the percentage of the population eventually infected in Japan and the United States.

## Results

### Parameters and Prediction for Japan

A typical candidate set of parameter values that fit well with the data from January 15, 2020, to March 20, 2020, is as follows: 1.3941<*k_j_*^(c)^<1.5979; 0.0982<*α_j_*<0.1158; 0.3895 *β_j_*<0.5163; 0.0098<*γ_j_*^(^*^I^*^)^<0.0128; 0.0027<*γ_j_*^(^*^C^*^)^<0.0047; 0.0019<*λ_j_*<0.0052; *κ*=0.1861; *k_h_*=0.6514. This set of parameters reflects an inadequate level of control to slow the spread of the disease, as indicated by the value of *k_j_*^(c)^ being larger than 1. Specifically, for each additional infected or exposed individual, the number of eventual infected individuals would increase by around 1.5 on average. The number of individuals eventually infected will approach a saturating percentage *k_h_*.

**Figure 2:**
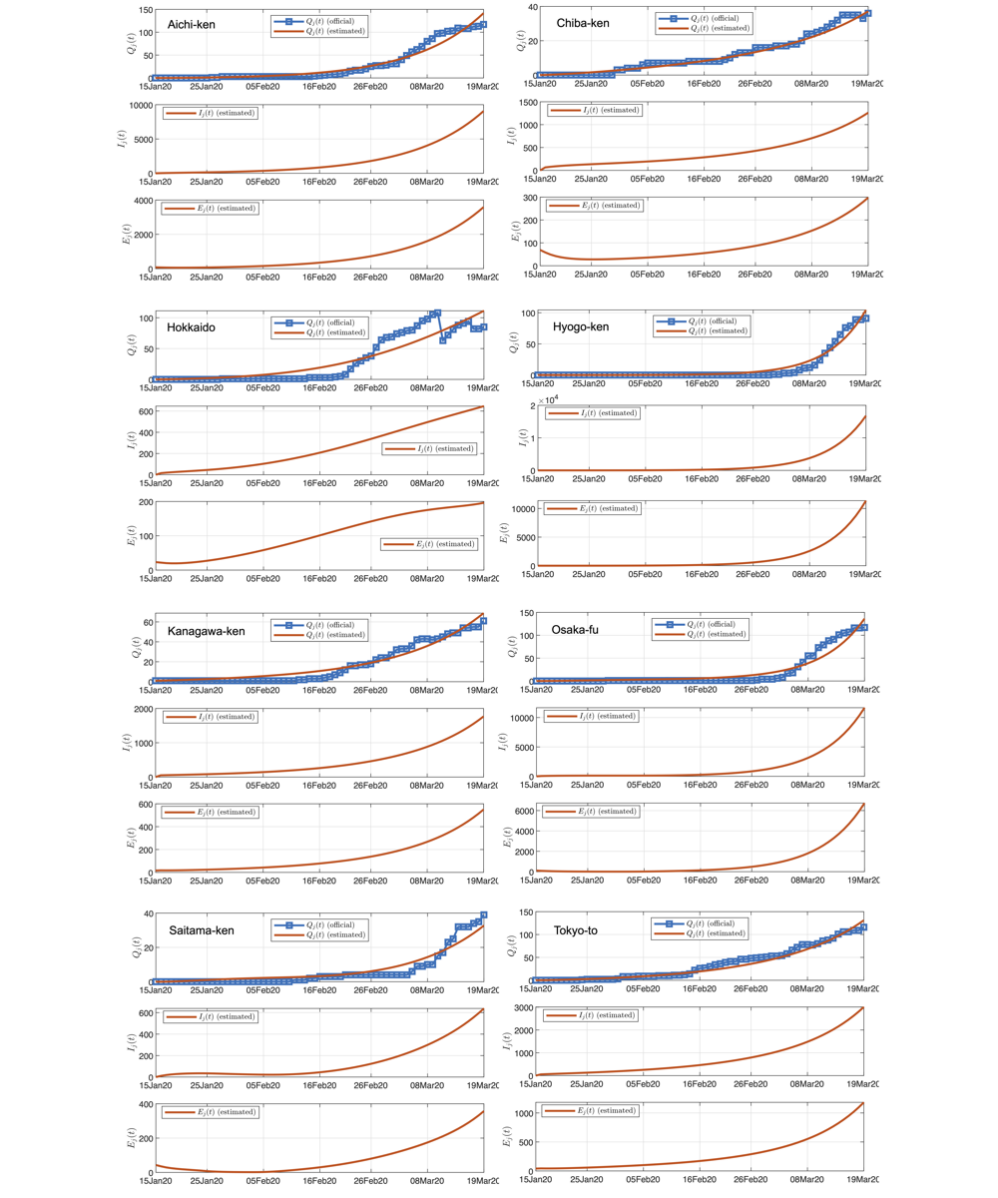
Official and estimated number of infected individuals in 8 selected prefectures in Japan (upper), the estimated number of infected individuals (not confirmed; middle), and the estimated number of exposed individuals (lower).

**Figure 3:**
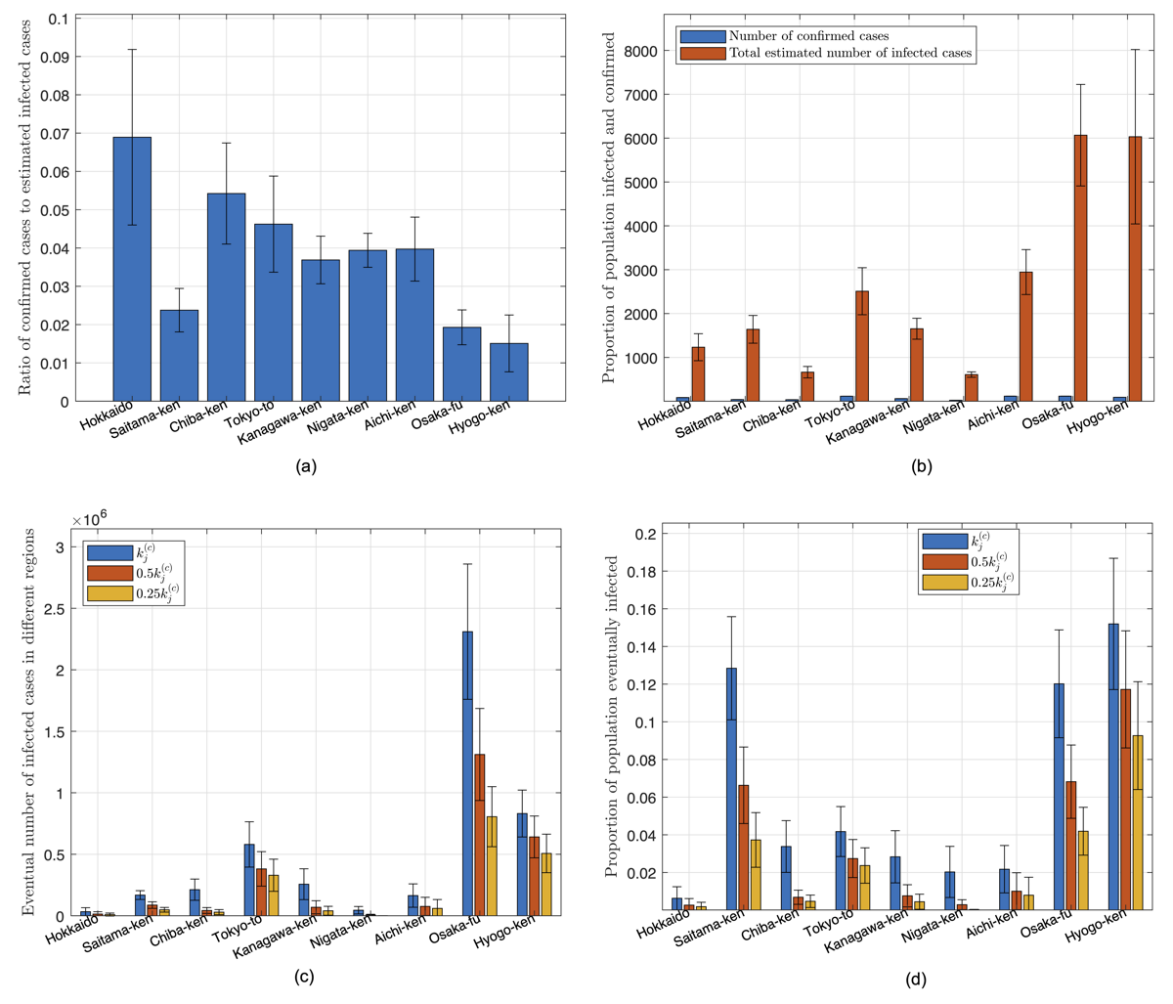
Statistics of data and predicted results for Japan. (a) Proportion of infected cases that are confirmed as of March 20, 2020; (b) number of confirmed cases and estimated number of infected cases as of March 20, 2020; (c) number of individuals eventually infected under three levels of active intervention; (d) proportion of population eventually infected under three levels of active intervention.

We have identified 100 candidate sets of parameters that satisfy the fitting criteria, and for each set of parameters, we perform a separate simulation run. Figure 2 shows one particular simulation run of a well-fitted candidate set of parameters for 8 selected prefectures in Japan. The averaged results of all simulation runs are consolidated in the charts shown in Figure 2. Based on the data up to March 20, 2020, our model estimates that less than 3% of the infected cases are confirmed, with Hokkaido having the highest percentage (6.9%) and Hyogo-ken the least (1.5%), as shown in Figure 3(a). In other words, the actual number of infected individuals could be 20 times as many as the official confirmed number. Statistics of percentages for the population of confirmed and infected with the disease up to March 20, 2020, are shown in Figure 3(b).

We examine three cases corresponding to the level of active intervention being unchanged, 2-fold elevated, and 4-fold elevated. First, staying with the status quo (*k_j_*^(c)^ unchanged), if there is no further tightening of control aiming to slow the spread, all parameters of the candidate sets will remain unchanged. The total number of individuals eventually infected until September 23, 2020, in each region is shown in Figure 3(c). In this case, the number of infected individuals in Osaka-fu and Tokyo-to will reach about 2,300,000 and 600,000 (12% and 4.2% of the population), respectively, while most other regions will have around 5% of the population eventually infected by September 23, 2020, as shown in Figure 3(d). In total, about 6.55% of the population in Japan will be infected.

Second, with two-fold elevated active intervention (*k_j_*^(c)^ → 0.5*k_j_*^(c)^), if active intervention is stepped up to twice the current level (ie, the value of *k_j_*^(c)^ is set to half of the original value in each simulation run), we observe a significant drop in the number of individuals eventually infected, as given in Figure 3(c). Specifically, the percentage of the population eventually infected by September 23, 2020, in Osaka-fu and Tokyo-to would drop to about 6.8% and 2.3%, respectively, while most other regions would drop to less than 2%, as shown in Figure 3(d). In total, about 4.14% of the population in Japan will be infected.

Third, with 4-fold elevated active intervention (*k_j_*^(c)^ → 0.25*k_j_*^(c)^), if active intervention is stepped up to four times the current level (ie, the value of *k_j_*^(c)^ set to a quarter of the original value in each simulation run), we observe a drastic drop in the number of individuals eventually infected, as given in Figure 3(c). Specifically, the percentage of the population eventually infected by September 23, 2020, in Osaka-fu and Tokyo-to would drop to about 4.1% and 2.3%, respectively, while most other regions would drop to less than 1%, as shown in Figure 3(d). In total, about 1.54% of the population in Japan will be infected.

In addition, our model estimates that the number of infected individuals could be 20 times as many as the currently confirmed number due to various reasons such as insufficient testing. Based on the data collected so far and assuming no further tightening of control, our model estimates about 6.65% of the population will be eventually infected, and a 4-fold elevation in control efforts may bring it down to 1.54% (about a 75% reduction) and end the pandemic sooner.

### Parameters and Prediction for United States

**Figure 4:**
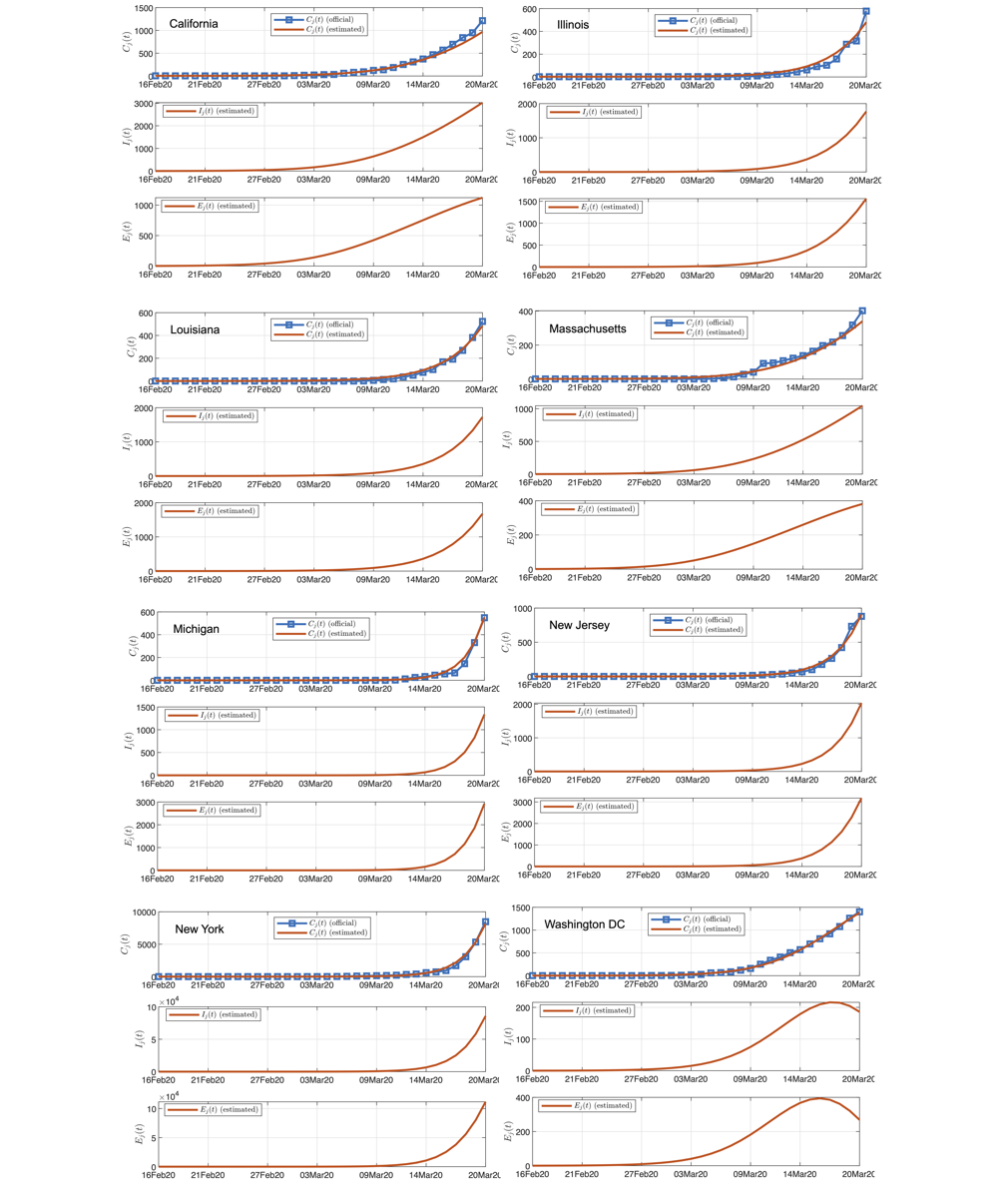
Official and estimated number of infected individuals in 8 selected states in the United States (upper), the estimated number of infected individuals (not confirmed; middle), and the estimated number of exposed individuals (lower).

We present here the results for eight selected states having significant numbers of infected individuals as of March 20, 2020. Figure 4 shows one typical simulation run, showing the number of confirmed cases, the estimated number of infected individuals (not confirmed), and the estimated number of exposed individuals. 

**Figure 5:**
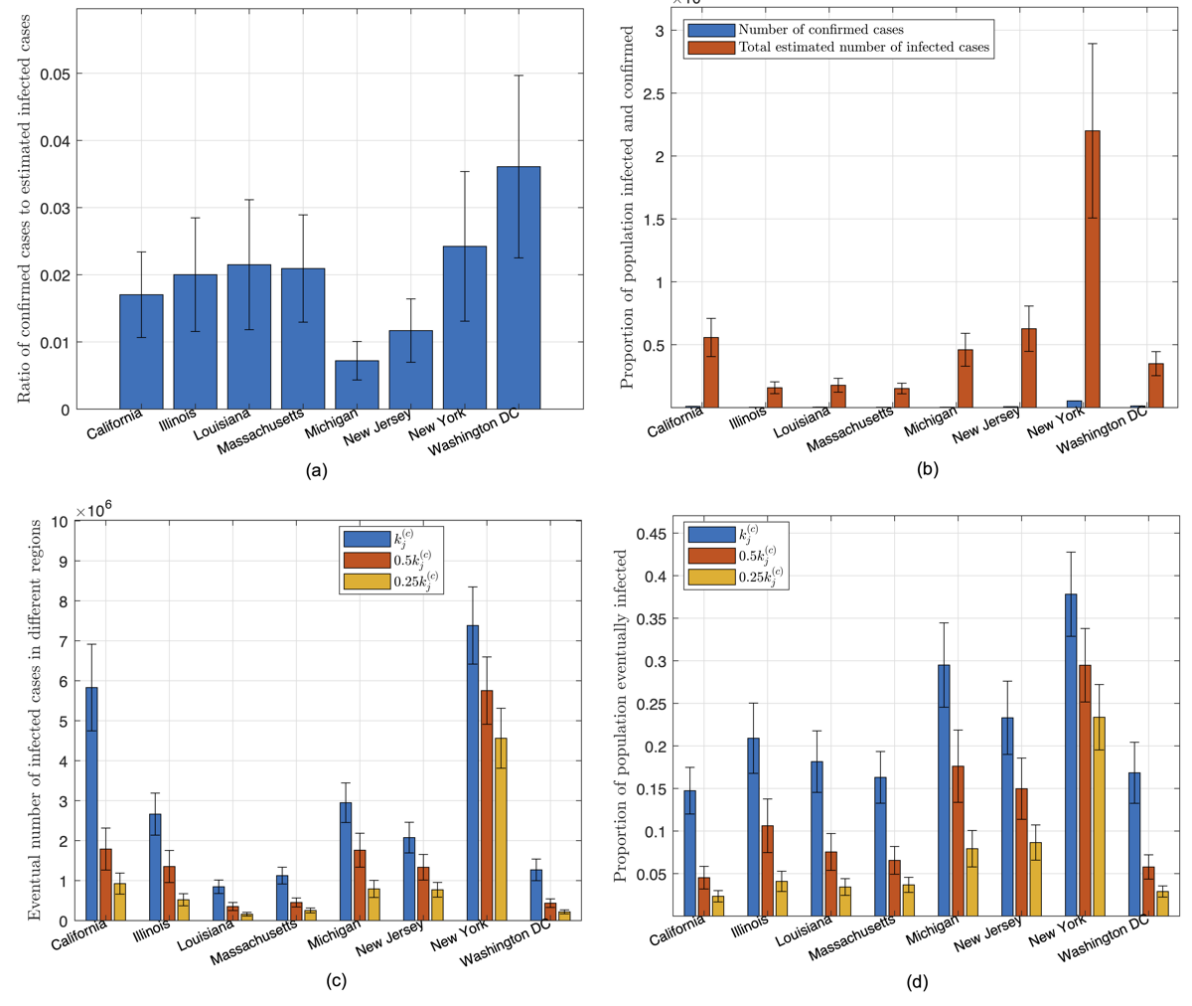
Statistics of data and predicted results for the United States. (a) Proportion of infected cases that are confirmed as of March 19, 2020; (b) number of confirmed cases and estimated number of infected cases as of March 19, 2020; (c) number of individuals eventually infected under three levels of active intervention; (d) proportion of population eventually infected under three levels of active intervention.

As of March 19, 2020, our model showed that less than 20% of the infected cases are confirmed, with Washington, DC having the highest percentage (36%) and Michigan state the least (0.7%), as shown in Figure 5(a). In other words, the actual number of infected individuals in the United States could be 5 times as many as the confirmed number. Statistics of percentages for the population of confirmed and infected with the disease up to March 19, 2020, are shown in Figure 5(b).

The key results for the three cases corresponding to three different levels of active intervention are as follows. First, staying with the status quo (*k_j_*^(c)^ unchanged), if there is no further tightening of control aiming to slow the spread, all parameters of the candidate sets will remain unchanged. The total number of individuals eventually infected until September 23, 2020, in each state is shown in Figure 5(c). In this case, the number of infected individuals in California and New York State will reach about 5,800,000 and 7,300,000 (15% and 37.5% of population), respectively, while most other states will have less than 20% of the population eventually infected by September 23, 2020, as shown in Figure 5(d). In total, about 18.2% of the population in the United States will be infected.

Second, with 2-fold elevated active intervention (*k_j_*^(c)^ → 0.5*k_j_*^(c)^), if active intervention is stepped up to twice the current level (ie, the value of *k_j_*^(c)^ set to half of the original value in each simulation run), we observe a significant drop in the number of individuals eventually infected, as given in Figure 5(c). Specifically, the percentage of the population eventually infected by September 23, 2020, in California and New York State would drop to about 4.5% and 29.5%, respectively, while most other states would drop to less than 10%, as shown in Figure 5(d). In total, about 14% of the population in the United States will be infected.

Third, with 4-fold elevated active intervention (*k_j_*^(c)^ → 0.5*k_j_*^(c)^), if active intervention is stepped up to four times the current level (ie, the value of *k_j_*^(c)^ set to a quarter of the original value in each simulation run), we observe further reduction in the number of individuals eventually infected, as given in Figure 5(c). Specifically, the percentage of the population eventually infected by September 23, 2020, in California and New York State would drop to about 2.5% and 23%, respectively, while most other states would drop to less than 3%, as shown in Figure 5(d). In total, about 9.32% of the population in the United States will be infected.

**Figure 6:**
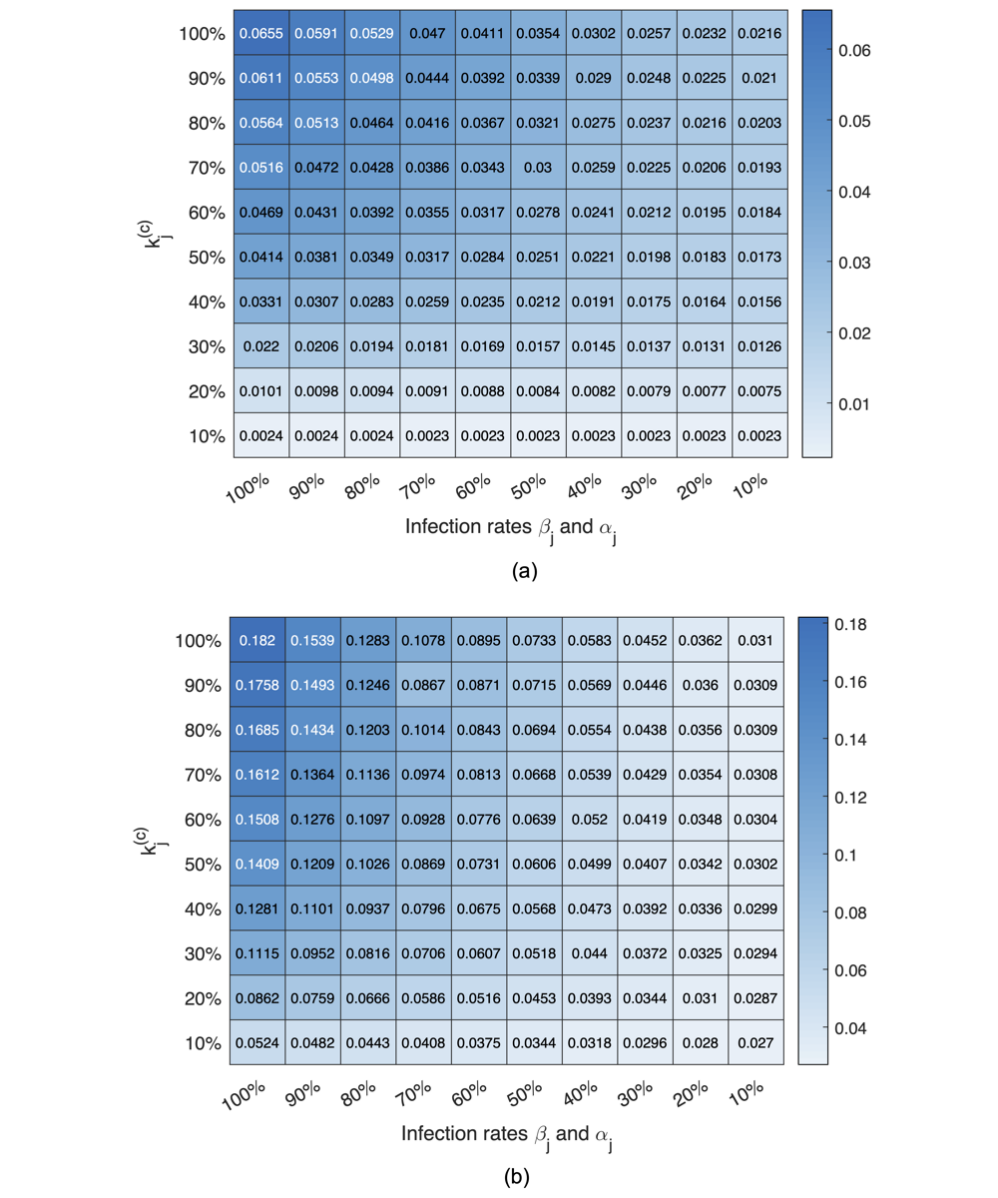
The proportion of the population eventually infected under different levels of the active intervention indicated by kj(c) (smaller the stronger) and maintaining personal hygiene and exercising protective measures indicated by αj, βj (smaller the stronger). (a) Japan; (b) the United States.

The results of assessing the combined effectiveness of active intervention and practicing protective measures in controlling the pandemic through adjusting parameters *α_j_*, *β_j_*, and *k_j_*^(c)^ are shown in Figure 6(a) and 6(b).

## Discussion

### Principal Findings

A significant step-up in the level of active intervention is necessary to slow the spread of the virus, especially for the United States. Based on the data collected up to March 20, 2020, and assuming no further tightening of the governments’ control, our model estimates that about 6.55% and 18.2% of the population would eventually be infected in Japan and the United States, respectively, and a drastic 10-fold elevated active control may bring it down further to 0.24% and 5.24% for Japan and the United States, respectively.

Our results have highlighted the ability of the model in assessing the impact of active intervention through adjusting one of the parameters, namely, *ψ_j_* = 1/*k_j_*^(c)^. Moreover, it has been widely disseminated that maintaining personal hygiene is equally important in curbing the spread of the virus. The World Health Organization recommends several specific protective measures to be practiced by the public including frequent hand washing; maintaining social distancing, avoiding touching one’s eyes, nose, and mouth; and practicing respiratory hygiene [21]. Recent studies have also shown that wearing surgical masks would help in some cases [22,23]. The level of public vigilance in exercising personal protective measures can also be incorporated in our model through adjusting infection rates *α_j_* and *β_j_*. We can, therefore, assess the combined effectiveness of active intervention and practicing protective measures in controlling the pandemic. Here, we varied *α_j_*, *β_j_*, and *k_j_*^(c)^ from 10% to 100% of the originally identified values in 10 intervals, corresponding to 10 different levels of public vigilance and active intervention by the authorities. In particular, we assess *α_j_* and *β_j_* as one property and *k_j_*^(c)^ as another (ie, varying *α_j_* and *β_j_* in synchrony). Specifically, for each candidate parameter set, we performed 100 simulation runs for each combination of *α_j_*, *β_j_*, and *k_j_*^(c)^, where *α_j_*, *β_j_*, and *k_j_*^(c)^ varied from 10% to 100% of the original values in 10 steps. We then investigated the percentage of the population eventually infected in Japan and the United States. The results are shown in Figure 6(a) and (b).

The mean percentage of the population eventually infected under different combinations of parameter values for Japan and the United States are given in the charts shown in Figure 6(a) and (b), respectively. For instance, suppose the level of public vigilance has dramatically raised and the level of active intervention has been stepped up, resulting in a 90% reduction in the infected rates and a 90% reduction in *k_j_*^(c)^ (ie, parameters changed to 0.1*α_j_*, 0.1*β_j_*, and 0.1*k_j_*^(c)^). Referring to Figure 6(a) and (b), the percentage of the population eventually infected can be dramatically reduced to 0.23% for Japan and 2.7% for the United States. Similar interpretations can be taken for any other combination of public vigilance levels and active intervention. 

Our results have highlighted an interesting difference between the effectiveness of government’s active intervention and maintaining personal hygiene by the public for Japan and the United States. For Japan, we observed a 27-fold reduction (from 6.55% to 0.24%) in the percentage of individuals eventually infected upon a drastic 10-fold step-up of active intervention (*k_j_*^(c)^ changed to 0.1*k_j_*^(c)^), whereas less than 3-fold reduction (from 6.55% to 2.16%) is observed in the percentage of individuals eventually infected upon the same 10-fold improvement in personal hygiene (values of *α_j_* and *β_j_* reduced by a factor of 0.1). Thus, government’s active intervention seems to be more important for Japan. Moreover, for the United States, we see the opposite. Specifically, only about 4-fold reduction in the percentage of individuals eventually infected is observed upon a drastic 10-fold step-up of active intervention, whereas a 6-fold reduction is observed upon a 10-fold improvement in maintaining personal hygiene by the public. Thus, raising the level of public vigilance in exercising personal protective measures is comparatively more important for the United States. The reason for the difference between Japan and the United States is that the United States has higher infection rates compared to Japan. Reducing *k_j_*^(c)^ for the US case is thus less effective at such high infection rates (ie, a smaller eventual infected number per additional infected individual would not help too much). In contrast, the parameter sets for Japan already have relatively lower infection rates, and further improvement by reducing the infection rates would be limited. As a final remark, combining a very high level of pubic vigilance in exercising strict protective measures and a drastic step-up of government intervention, the percentage of the population getting infected can be reduced to 0.23% in Japan and 2.7% in the United States.

As this study was conducted during the early phase of the pandemic for Japan and the United States, the amount of data used was moderate, though adequate in generating sets of parameter values that fit the data with sufficiently small errors. With more data available, the accuracy of the parameters obtained is expected to improve, and the prediction henceforth would also be more accurate. However, in predicting the pandemic progression, especially during the early phase of an outbreak, we are often confronted with limited data, and the results in this study did demonstrate the application of the proposed model and data fitting method in offering highly consistent prediction of the extent of the pandemic (eg, percentage of the population infected) for Japan and the United States.

Several limitations of the model presented here are worth noting. First, we observed that the actual epidemic trajectory deviates above or below the estimated trajectory due to the varying levels of public health measures applied at particular times, which cause parameters *k_j_*^(c)^, *α_j_,* and *β_j_* to vary with time. Thus, if a city or region has implemented highly successful public health measures, then the actual values of *k_j_*^(c)^, *α_j_,* and *β_j_* would be less than their estimated values. The number of confirmed cases would be less than that estimated by the model and vice versa. Furthermore, the number of confirmed cases is highly related to the number of patients who have been tested [23,24]. The value of *λ_j_* is thus also time varying as the test capacity varies in time. In our model, we take the parameters as constants for simplicity. Using constant parameters, the model can only give an average profile prediction. Second, expanding the parameter set would improve the ability of the model to isolate the different causes that contribute to the pandemic progression profile. For instance, we may introduce a parameter corresponding to the testing capacity of a city or region instead of integrating it with *λ_j_*, which may blur the key factor affecting *λ_j_*. However, with more parameters, the parameter extraction process will become more time-consuming and computationally more intensive. Thus, a right balance should be sought to achieve an adequate coverage of interpretation for physical causes by the parameter set while maintaining a reasonable computational efficiency. Finally, the model has a large set of parameters, and the relative importance of each parameter is not identical [25,26]. A detailed sensitivity analysis can be performed to identify the set of crucial parameters so that resources can be directed to specific kinds of active measures to slow the pandemic progression more effectively.

### Conclusion

One of the key challenges in data-driven modelling and analysis is the delayed and missing information that makes fitting of models either difficult or unreliable, resulting in inconsistent or even erroneous dynamical profiles generated by a poorly parameterized model. The traditional SEIR model provides a general dynamical description of the disease spread in a population and involves a series of transitional processes that describe how a healthy individual becomes exposed, infected, and eventually recovered or removed from the population. However, the data of infected and recovered cases reported by different cities and regions have been found unreliable or incomplete, as they are subject to the availability of test facilities as well as other factors related to the bureaucracy of reporting and the operation mode of the medical systems. In this paper, we propose a new disease spreading model with consideration of the delayed and missing data of infected cases, intercity travel, and the level of active intervention. The model, which estimates the actual number of infected cases after identifying the best parameter sets, was applied to study the COVID-19 pandemic progression in Japan and the United States. Results reveal that the actual number of infected individuals could be up to 20-fold and 10-fold as many as the confirmed numbers in Japan and the United States, respectively, as of March 19, 2020. Our model also allows assessment of varying levels of active intervention implemented by the government, and the results showed that the current level of control by the Japanese and US governments may be inadequate, and a significant step-up in the level of active intervention is necessary to slow the aggressive progression trend in both countries. For Japan, based on the data collected so far and assuming no further tightening of control, our model estimates about 6.55% of the population eventually infected, and a 4-fold elevation in control efforts may bring it down to 1.54%. For the United States, our model estimates about 18.2% of population will eventually be infected if the government does not step up its control, and a 4-fold elevation in active intervention may bring it down to 9.32%. Finally, adjusting the infection rates permits assessing the effectiveness of practicing protective measures and maintaining personal hygiene. Our results show that stepping up government’s active intervention would be more effective for Japan, while raising the level of public vigilance in maintaining personal hygiene and social distancing is comparatively more important for the United States.

## Abbreviations

C: confirmed
COVID-19: coronavirus disease
E: exposed
I: infected
R: recovered or removed
S: susceptible
SEICR: susceptible-exposed-infected-confirmed-removed
SEIR: susceptible-exposed-infectious-recovered
